# Profiles of impulsivity and alcohol use: Unveiling personality, cognitive traits, and DSM diagnoses

**DOI:** 10.1111/acer.70116

**Published:** 2025-08-07

**Authors:** Chloe Lau, Danielle Downie, R. Michael Bagby, Bruce G. Pollock, Anthony C. Ruocco, Lena C. Quilty

**Affiliations:** ^1^ Campbell Family Mental Health Research Institute Centre for Addiction and Mental Health Toronto Ontario Canada; ^2^ Department of Psychiatry, Schulich Medicine and Dentistry Western University London Ontario Canada; ^3^ Graduate Department of Psychological Clinical Science University of Toronto Toronto Ontario Canada; ^4^ Department of Psychology University of Toronto Scarborough Toronto Ontario Canada; ^5^ Department of Psychiatry University of Toronto Toronto Ontario Canada

**Keywords:** alcohol, cognition, impulsivity, personality, profiles

## Abstract

**Background:**

Impulsivity is closely associated with alcohol use, but limited research has explored distinct latent profiles encompassing impulsivity traits and alcohol use disorder symptoms.

**Methods:**

This study used latent profile analysis (LPA) to investigate these patterns among 201 adult outpatients (50% female, 50% male) from a tertiary care setting. Participants completed self‐reported measures such as the Alcohol Use Disorders Identification Test (AUDIT), Impaired Control Scale, and UPPS‐P Impulsivity Scale, as well as performance‐based tasks like the Probability Reward Task (PRT) and Stop Signal Reaction Time Task.

**Results:**

LPA identified three profiles using AUDIT, impaired control, and UPPS‐P: (1) Low‐Risk Profile—characterized by low levels of alcohol use disorder (AUD) symptoms and impulsivity; (2) Emotionally Reactive Profile—characterized by elevated impulsivity with low AUD symptoms; and (3) High‐Risk Profile—characterized by elevated levels of both AUD symptoms and impulsivity. ANCOVA results revealed that Emotionally Reactive individuals scored higher on neuroticism, negative affectivity, and psychoticism and lower on conscientiousness compared to the Low‐Risk group. Both Emotionally Reactive and High‐Risk groups showed lower agreeableness, antagonism, and disinhibition relative to the Low‐Risk group. On cognitive tasks, the Low‐Risk group outperformed the High‐Risk group in PRT accuracy and discriminability, while Emotionally Reactive and Low‐Risk groups showed similar advantages over High Risk.

**Conclusions:**

These findings reveal distinct personality and cognitive profiles linked to reward and control processes, informing tailored interventions for impulsivity and alcohol‐related harms.

## Graphical abstract

This study identified three latent profiles of alcohol use disorder symptoms (AUDS) and impulsivity among 201 adult outpatients: Low Risk (low AUDS and impulsivity), Emotionally Reactive (high impulsivity with low AUDS), and High Risk (high AUDS and impulsivity). Profiles differed in broad and maladaptive personality traits, as well as cognitive task performance (e.g., probabilistic reward task). These findings reveal distinct risk profiles vulnerable to impulsivity‐related alcohol use challenges.

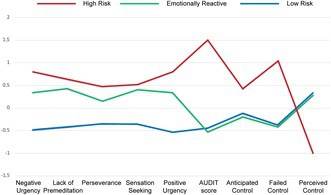

## INTRODUCTION

Alcohol‐related harms are strongly linked to impulsivity, as impulsive decision making often undermines self‐control and increases the risk of alcohol dependence (Dick et al., 2010; Mitchell et al., 2013; Stamates & Lau‐Barraco, 2017; Wilson et al., 2024). Impulsivity encompasses various traits, including acting without sufficient forethought or displaying tendencies toward rash, unintended reactions without considering potential negative outcomes (Moeller et al., 2001; Vassileva & Conrod, 2019). Importantly, impulsivity is frequently highlighted in the Diagnostic and Statistical Manual of Mental Disorders (DSM) as a key characteristic of substance use disorders, particularly those involving drug‐seeking behaviors (American Psychiatric Association [APA], 2013; Carvalho et al., 2019; Whiteside & Lynam, 2001). Additionally, impulsivity has been associated with poorer treatment responsiveness (Barratt et al., 1997) and less favorable outcomes, as individuals with high impulsivity often exhibit greater resistance to treatment and increased risk of relapse compared to those with lower impulsive traits (Hershberger et al., 2017; Loree et al., 2015). Thus, impulsivity represents a critical factor in the treatment and management of individuals with psychiatric disorders, specifically within the context of alcohol use.

To capture the complexity of impulsivity, researchers frequently utilize the UPPS‐P Impulsive Behavior Scale, a widely accepted multidimensional model of impulsivity assessing five distinct dimensions: negative urgency, positive urgency, lack of premeditation, lack of perseverance, and sensation seeking (Cyders, 2013; Vassileva & Conrod, 2019). Negative and positive urgency represent emotion‐driven aspects of impulsivity and are positively associated with both low‐risk and problematic alcohol use (Coskunpinar et al., 2013; Smith & Cyders, 2016; Stautz & Cooper, 2013). These constructs are closely tied to risky behaviors, as individuals with higher urgency scores often demonstrate poorer emotional regulation (Cyders & Smith, 2008; Settles et al., 2010; Stautz et al., 2017). Lack of premeditation, reflecting a tendency to act without prior planning or consideration of potential consequences, has been linked to higher rates of heavy alcohol consumption and alcohol use disorders (Adams et al., 2012; Carlson et al., 2010; Lynam & Miller, 2004; Verdejo‐García et al., 2007). Sensation seeking, characterized by pursuing novel and potentially risky experiences, shows strong associations with heavy alcohol use, particularly among young adults and women (Carlson et al., 2010; Evans‐Polce et al., 2018; Horvath et al., 2004; Magid & Colder, 2007; Puente et al., 2008; Settles et al., 2010). Lack of perseverance, or difficulty maintaining focus on tedious or challenging tasks, demonstrates mixed associations with alcohol use (Hopwood & Sellbom, 2013; Kozak et al., 2019). Collectively, these distinct facets robustly predict problematic alcohol use, with the UPPS‐P Impulsive Behavior Scale capturing both shared and unique contributions of each dimension (Adams et al., 2012).

Despite considerable research on impulsivity, limited work has adopted latent profile analysis (LPA) to explore how combinations of impulsivity facets relate to alcohol use outcomes. Latent profile analysis is a person‐centered analytical approach that classifies individuals into subgroups based on shared characteristics. Prior studies employing LPA have identified profiles varying in impulsivity traits and associated alcohol outcomes. For example, Stamates et al. (2021) applied LPA using UPPS‐P facets, identifying profiles characterized by uniformly high impulsivity associated with greater alcohol‐related negative consequences but not necessarily increased drinking behaviors. Extending this work, Stamates et al. (2023) analyzed a sample engaging in simultaneous alcohol and marijuana use, uncovering a four‐profile solution where a profile high in sensation seeking and urgency was associated with higher alcohol use disorder severity and past‐year substance use compared to lower impulsivity profiles. Similarly, Rogers et al. (2021) found that profiles with pronounced deficits in premeditation and perseverance correlated with elevated alcohol consumption.

Previous research using LPA highlights substantial variability among individuals who consume alcohol (Kuvaas et al., 2014). For instance, identified profiles have ranged from light to moderate, heavy, and problematic drinkers, with emotional self‐regulation and urgency distinguishing high alcohol use disorder (AUD) symptoms compared to low AUD symptoms (Kuvaas et al., 2014). Additionally, profiles marked by heavy or problematic drinking often cooccur with severe psychopathology, such as social anxiety, attention‐deficit hyperactivity disorder (ADHD), and posttraumatic stress disorder (PTSD) (Campbell et al., 2021; Merrill et al., 2020; Villarosa‐Hurlocker & Madson, 2020).

Further exploration into the interplay between impulsivity and alcohol use via LPA is essential to clarify distinct subgroup characteristics and associated outcomes. While impulsivity is a well‐established risk factor for alcohol use disorder, not all individuals with elevated impulsivity engage in problematic alcohol use or experience associated harms (Herman & Duka, 2019). Identifying the factors distinguishing impulsive individuals who develop problematic alcohol use from those who do not is therefore crucial. Given the predictive role of impulsivity in the initiation, persistence, and relapse of substance use, differentiating impulsivity‐based profiles can enhance our understanding of individual variability in alcohol‐related outcomes and inform tailored treatment approaches (Smith & Cyders, 2016).

This study advances the literature on impulsivity and alcohol use by integrating self‐reported impulsivity traits and AUD symptoms into a unified latent profile analysis. While prior studies have independently explored UPPS‐P traits or alcohol misuse patterns, few have adopted a person‐centered, multimodal approach incorporating both clinician‐administered diagnostic interviews and performance‐based cognitive tasks. By identifying distinct subgroups, including those with differing impulsivity and drinking profiles, the findings challenge the assumption that impulsivity uniformly leads to alcohol misuse. This contributes important nuance to the understanding of heterogeneity in impulsivity‐related risk and highlights potential targets for more tailored prevention and intervention strategies, particularly for individuals with high impulsivity but no current alcohol‐related harms. This approach aligns with calls in the field for dimensional, transdiagnostic models that bridge personality, cognition, and clinical outcomes (Koudys et al., 2023). This person‐centered approach is especially valuable in this context, as it allows for a nuanced examination of the complex, multivariate relationships between impulsivity traits, cognitive functioning, and clinical outcomes.

### Objectives

The study aimed to address the following research questions:

**Aim 1:** To identify latent profiles based on patterns of alcohol use and impulsivity traits.

Hypothesis 1 Distinct latent profiles will emerge, such as a high‐risk profile characterized by elevated alcohol use and impulsivity, and a low‐risk profile with minimal alcohol use and impulsivity.

**Aim 2:** To examine the associations between the extracted profiles and normative/pathological personality traits, as well as behavioral activation and inhibition systems.

Hypothesis 2 Profiles characterized by higher alcohol use and impulsivity will be associated with elevated levels of maladaptive personality traits (e.g., disinhibition, antagonism), heightened behavioral activation system sensitivity, and reduced behavioral inhibition system sensitivity.

**Aim 3:** To investigate whether the identified profiles differ in performance‐based measures of impulsivity.

Hypothesis 3 Profiles with higher self‐reported impulsivity will show greater deficits on behavioral tasks measuring impulsive decision making and response inhibition.

## METHODS

### Sample and procedure

Participants were recruited through the hospital research registry. All participants were contacted regarding this research opportunity and, following a brief telephone screening interview, were invited to complete two research assessment visits. Of the 871 individuals who received a study overview, 354 completed the telephone screen. Among those, 279 were deemed eligible, and 201 attended the research lab, provided oral and written consent after a full explanation of the study procedures, and completed the measures outlined below. All measures were administered according to the study protocol and in full compliance with the ethical standards and standard operating procedures of this well‐established psychiatric hospital and academic health science center. Eligibility criteria included the presence of current, clinically significant psychiatric symptoms, attendance at a hospital appointment within the past 12 months, and consent to join the research registry. Exclusion criteria were severe homicidal or suicidal ideation, a current psychotic disorder, or current intoxication or withdrawal.

The final sample included 201 outpatients (50% male, 50% female) aged 18 to 87 years (*M* = 39.66, SD = 13.76). All participants reported clinically significant psychiatric symptoms and had attended a treatment appointment at the hospital within the past year. The sample reflected a diverse range of racial and ethnic backgrounds: Asian/Pacific Islander (9%), Black (3%), European White (76%), First Nations (4%), Latin American (5%), and other or multiracial (5%).

Licensed clinical psychologists administered the Structured Clinical Interview for DSM‐IV Patient Version (SCID‐I/P) to determine clinical diagnoses based on DSM criteria (First et al., 1995; First & Gibbon, 2004). While individual diagnoses were assigned to each participant, for data analysis purposes, they were grouped into three categories: depressive disorders, anxiety disorders, and substance use disorders. Diagnoses attributed to general medical conditions or substance‐induced effects were excluded. Among those who provided consent and met eligibility criteria, 145 participants (72.5%) met diagnostic criteria for a lifetime mood disorder, and 66 (33%) met criteria for a current mood disorder. Additionally, 109 participants (53%) met criteria for a lifetime substance use disorder, and 38 (18%) for a current substance use disorder. Anxiety disorders were defined as any DSM diagnosis of panic disorder, agoraphobia without panic disorder, social anxiety disorder, specific phobia, obsessive‐compulsive disorder, posttraumatic stress disorder, generalized anxiety disorder, or anxiety disorder not otherwise specified. In this sample, 98 participants (49%) met criteria for a lifetime anxiety disorder, and 64 (32%) for a current anxiety disorder.

### Latent profile analyses measures

#### Alcohol use disorder symptoms

The Alcohol Use Disorders Identification Test (AUDIT) is a widely used self‐report screening measure designed to identify individuals with hazardous alcohol consumption patterns or potential alcohol dependence. Developed by the World Health Organization (WHO), the AUDIT comprises 10 items covering three key domains: (1) Alcohol consumption patterns (frequency and quantity), (2) Drinking behaviors (such as binge drinking), and (3) Alcohol‐related problems (including dependence symptoms and alcohol‐related harms). Responses are scored on a scale from 0 to 4, with higher total scores indicating greater severity of alcohol‐related risk. A score of 8 or more typically signals hazardous drinking behaviors or potential alcohol use disorders (Saunders et al., 1993). The AUDIT has demonstrated strong validity and reliability across diverse populations and settings, making it an internationally recognized tool for both clinical screening and research applications. In this study, the AUDIT shows strong reliability, with a Bayesian McDonald's *ω* posterior mean of 0.90 (95% Credible Intervals [CIs]: 0.88, 0.92) with a noninformative prior. Cronbach's *α* is reported as 0.90.

#### Impaired control

The Impaired Control Scale consists of 25 items designed to measure three aspects of control related to alcohol use: attempted control (efforts to limit alcohol consumption), failed control (inability to regulate drinking), and perceived control (difficulty in limiting alcohol use). Responses are recorded on a five‐point Likert scale ranging from 0 (never) to 4 (always), with “not applicable” responses scored as 0 (Heather et al., 1993, 1998). The scale demonstrates strong reliability, with Bayesian McDonald's *ω* posterior means ranging from 0.71 to 0.94 (*α* = 0.74 to 0.95).

#### Impulsivity

The UPPS‐P Impulsive Behavior Scale assesses five dimensions of impulsivity: perseverance, premeditation, sensation seeking, positive urgency, and negative urgency. Responses are captured on a four‐point Likert scale ranging from 1 (strongly disagree) to 4 (strongly agree) (Cyders et al., 2007). The theoretical framework is a widely established model for impulsivity (Hershberger et al., 2017). The scale demonstrates high reliability, with Bayesian McDonald's *ω* posterior means ranging from 0.80 to 0.92 and Cronbach's *α'*s ranged from 0.79 to 0.95. Additionally, the five subscales have shown strong evidence of both factorial and criterion validity across various assessment methods (Cyders & Smith, 2008; Smith et al., 2007).

### External validity measures

#### Big Five personality

The Big Five Aspects Scales (BFAS) is a 100‐item self‐report questionnaire that assesses five personality traits: openness to experience, conscientiousness, extraversion, agreeableness, and neuroticism. Responses are recorded on a 5‐point Likert scale, ranging from 1 (very inaccurate) to 5 (very accurate) (DeYoung et al., 2007). The five‐factor structure has demonstrated strong evidence of factorial validity, internal consistency, and external validity (DeYoung et al., 2007). Reliability for the five traits, measured by McDonald's *ω*, ranges from 0.84 to 0.91 (Table 1 provides detailed values) and subscale values of Cronbach's *α* ranged from 0.77 to 0.90.

**TABLE 1:** Means, standard deviations, reliabilities, and bivariate bayesian pearson's rho correlations between alcohol use harms, impulse control, and impulsivity.

	Mean (SD)	Alcohol use harms	Anticipated control	Failed control	Predicted control	Negative urgency	Lack of premeditation	Lack of perseverance	Sensation seeking	Positive urgency
Alcohol use harms	7.56 (8.30)	0.*90* [0.88, 0.92]								
Anticipated control	9.07 (7.53)	0.29***	*0.94* [0.93, 0.95]							
Failed control	12.23 (7.68)	0.59***	0.17	*0.71* [0.65, 0.77]						
Predicted control	28.02 (11.19)	−0.58***	−0.20	−0.58***	*0.93* [0.91, 0.94]					
Negative urgency	2.70 (0.65)	0.26**	0.13	0.31***	−0.23*	*0.87* [0.84, 0.90]				
Lack of premeditation	2.07 (0.54)	0.20	0.02	0.14	−0.05	0.41***	*0.83* [0.79, 0.86]			
Lack of perseverance	2.43 (0.54)	0.16	0.01	0.14	−0.06	0.30***	0.29***	*0.80* [0.75, 0.84]		
Sensation seeking	2.48 (0.71)	0.12	0.03	−<0.01	−0.07	0.30***	0.40***	0.10	*0.86* [0.83, 0.89]	
Positive urgency	2.21 (0.80)	0.26**	0.12	0.25**	−0.27***	0.63***	0.34***	0.28***	0.34***	*0.92* [0.90, 0.93]

*Note*: Bayesian Scale Reliability McDonald's Omega posterior means are listed in the diagonal in italics with 95% Credible Interval (CI) lower bound and upper bound. *BF_10_ > 10, **BF_10_ > 30, ***BF_10_ > 100 for Bayesian Pearson's rho correlations.

#### Pathological personality

The Personality Inventory for DSM‐5 (PID‐5) is a 220‐item self‐report measure designed to evaluate five maladaptive personality traits: negative affect, detachment, antagonism, disinhibition, and psychoticism. Responses are recorded on a 4‐point Likert scale ranging from 0 (very false or often false) to 3 (very true or often true) (Krueger et al., 2012; Quilty et al., 2013). The reliability of the five traits, as indicated by McDonald's *ω* posterior means, ranges from 0.81 to 0.88. The five‐factor structure has demonstrated strong reliability and validity across various studies (Al‐Dajani et al., 2016; Hopwood & Sellbom, 2013; Krueger & Markon, 2014; Quilty et al., 2013). Cronbach's *α* ranged from 0.76 to 0.96.

#### Behavioral activation and inhibition

The BIS/BAS scales were used to assess behavioral inhibition (BIS) and behavioral activation (BAS). The instrument consists of 20 items rated on a four‐point Likert scale ranging from 1 (very true for me) to 4 (very false for me). The BAS is divided into three subdomains: BAS Drive (motivation to achieve goals), BAS Reward Responsiveness (sensitivity to rewarding stimuli in the environment), and BAS Fun Seeking (motivation to pursue novel and spontaneous rewards). The scales have demonstrated strong reliability and validity across various studies (Carver & White, 1994). Cronbach's *α* ranged from 0.73 to 0.80.

#### Balloon Analogue Risk Task (BART)

The Balloon Analogue Risk Task (BART) is a computerized measure of risk‐taking behavior, with higher scores indicating a greater tendency for risk‐taking (Ashenhurst et al., 2014). During the task, participants interact with 90 balloons of three different colors, each associated with varying probabilities of popping. Participants can choose to “pump” the balloon to increase potential earnings or “bank” the accumulated money at any point. However, if the balloon pops before the money is banked, the participant loses the earnings for that balloon. Two key metrics are calculated: the average number of pumps for balloons that do not pop (adjusted average pumps; Lejuez et al., 2002) and the total number of balloons that pop (Reed et al., 2012).

#### Go‐No‐Go Task

The Go/No‐Go task is a computerized assessment used to measure response inhibition and reward sensitivity, with impulsive individuals often showing higher rates of commission errors or inhibition failures (Hamidovic et al., 2008). Participants are instructed to respond to specific stimuli (target “go‐stimuli”) while withholding responses to others (nontarget “no‐go stimuli”; Fillmore et al., 2006). In the modified version by Fillmore et al. (2006), a black rectangle outline initially appears, which then changes to green (“go‐stimuli”) or blue (“no‐go stimuli”) after a delay. The rectangle's orientation (horizontal or vertical) provides a probabilistic cue about the upcoming stimulus type. Performance is evaluated through two primary measures: (1) commission errors (responding to a no‐go stimulus) and (2) reaction time (Fillmore et al., 2006; Hamidovic et al., 2008).

#### Stop Signal Reaction Time Task (SSRT)

The Stop Signal Reaction Time (SSRT) task assesses the ability to inhibit an initiated response, illustrating the relationship between psychological processes and real‐time refractory periods (Lappin & Eriksen, 1966). The task involves two auditory stimuli: a consistent “go signal” presented in every trial and an unpredictable “stop signal” that occurs intermittently. Participants are instructed to suppress their planned response as quickly and accurately as possible upon hearing the stop signal (Lappin & Eriksen, 1966; Verbruggen & Logan, 2008). The task becomes more challenging as the time interval between the primary task stimulus and the stop signal decreases.

#### Probabilistic Reward Task (PRT)

The Probabilistic Reward Task (PRT) assesses an individual's ability to adjust behavior based on rewards (Pizzagalli et al., 2008). Each trial begins with a fixation period, followed by the presentation of a cartoon face without a mouth for 500 ms. Subsequently, a face with either a short or long mouth is displayed, and participants must identify which one appeared by pressing one of two keys. In each trial, only a subset of correct responses receives reward feedback. While participants are informed that rewards will be given for some correct responses, the uneven probability of rewards is not disclosed. If a response is incorrect in a trial scheduled for feedback, the reward is delayed until the next correct identification of the same stimulus type. This setup may lead to response biases, as participants may develop a preference for identifying the stimulus linked to more frequent rewards. The task consists of three blocks, each containing 100 trials and lasting approximately 8 min, with short breaks between blocks.

### Statistical analyses

The AUDIT, ICS (i.e., attempted control, failed control, perceived control), and UPPS‐P scales (perseverance, premeditation, sensation seeking, positive urgency, and negative urgency) were utilized to derive latent profiles, providing a comprehensive assessment of traits related to alcohol use and impulsivity. Table 1 includes the means, standard deviations, Bayesian Pearson's rho correlations, and Bayesian single‐test McDonald's *ω* values for these measures. Profile indicators were estimated using standardized *z*‐scores (Diallo et al., 2016), and the profiles were identified through the expectation–maximization (EM) algorithm for maximum likelihood estimation.

Profile selection was guided by specific criteria. First, models were evaluated using sample size‐adjusted BIC (SABIC), Bayesian Information Criterion (BIC), Akaike Information Criterion (AIC), and the significance of the Bootstrapped Likelihood Ratio Test (BLRT), with lower values preferred (Nylund et al., 2007; Spurk et al., 2020; Weller et al., 2020). Second, an entropy index greater than 0.80 was required to ensure clear profile separation (Celeux & Soromenho, 1996; Wang et al., 2017). Lastly, profiles needed to represent at least 5% of the sample and include over 25 cases to avoid overfitting and spurious results (Lubke & Neale, 2006; Spurk et al., 2020; Weller et al., 2020). If model fit criteria were inconclusive, the most parsimonious model aligned with theoretical frameworks was selected (Spurk et al., 2020; Weller et al., 2020).

The most and least significant variables were identified based on their ability to differentiate profiles using mean values. ANCOVAs, controlling for age and gender, were conducted to calculate effect sizes (*η* ^2^ and *ηp* ^2^) and Vovk–Sellke Maximum *p*‐Ratios (VS‐MPR), which represent the maximum odds favoring differences between groups over no differences between groups (Sellke et al., 2001). The extracted profiles were compared across various outcomes, including personality traits and performance‐based cognitive and impulsivity measures.

Mean differences were examined across profile memberships using Frequentist and Bayesian ANCOVA methods, controlling for age and gender (Rouder et al., 2012; van den Bergh et al., 2020; Wagenmakers et al., 2018). Default multivariate Cauchy priors were applied, with prior odds adjusted for multiple comparisons as recommended by van den Bergh et al. (2020) and Westfall et al. (1997). Bayes factors were interpreted as follows: 1 (no evidence), 1–3 (anecdotal evidence), 3–10 (moderate evidence), 10–30 (strong evidence), 30–100 (very strong evidence), and > 100 (extreme evidence) (Jeffreys, 1961; Lee & Wagenmakers, 2014; Stefan et al., 2019). When referencing Bayesian statistics results, P[M|Data] denotes the probability of the model given the data. P(M) = 0.13 in the null model with membership, gender, and age entered into the model. In the Bayesian analyses referenced in the subscript, M denotes membership, G denotes gender, and A denotes age.

Frequentist chi‐square tests and Bayesian multinomial tables assessed associations between profile membership and DSM diagnoses. Standardized residuals >1.96 indicated rejection of the null hypothesis (*p* = 0.05).

Psychiatric diagnoses were not included as indicators in the latent profile analysis but were incorporated into subsequent analyses examining profile correlates. Specifically, chi‐square tests and Bayesian multinomial models were used to evaluate associations between profile membership and lifetime DSM‐based diagnoses, including mood disorders, anxiety disorders, and substance use disorders. This approach allowed for the assessment of the relationship between psychiatric comorbidity and latent profiles post hoc, without influencing the profile derivation process.

Descriptive and Bayesian statistics were performed using JASP (v. 0.16.4), and latent profile analyses were conducted with Jamovi (v. 2.3) using the snowRMM module (Rosenberg et al., 2018; The Jamovi Project, 2025).

## RESULTS

### Extracted profiles

Table 1 shows associations between profile variables, and Table 2 presents the latent profile fit indices, class sizes, and interpretability. The AIC, BIC, and SABIC values decreased as additional profiles were extracted, with all profiles showing an entropy value >0.80. Solutions with more than three profiles were excluded due to smaller sample sizes (*n* < 25), making the three‐profile model the most suitable.

**TABLE 2:** Model fit indices, class size, and class probabilities for latent profile membership.

Profiles	AIC	BIC	SABIC	BLRT (*p*)	Entropy	*n* min	*n* max	Prob_min	Prob_max
Two‐profile	4713	4804	4715	249.88 (0.01)	0.814	0.4197	0.5803	0.9465	0.9509
**Three‐profile**	**4523**	**4646**	**4526**	**210.22 (0.01)**	**0.884**	**0.1451**	**0.5855**	**0.8950**	**0.9760**
Four‐profile	4471	4628	4476	71.12 (0.01)	0.867	0.1503	0.4145	0.9096	0.9758
Five‐profile	4435	4624	4440	56.49 (0.01)	0.883	0.0622	0.3834	0.8285	0.9739
Six‐profile	4441	4663	4448	13.57 (0.66)	0.825	0.0622	0.2435	0.7584	0.9742

*Note*: Bolded values represent the selected three‐profile solution.

Abbreviations: AIC, Akaike Information Criterion; BIC, Bayesian Information Criterion; *n* max, proportion of participants designated to the largest profile (most probable profile membership); *n* min, proportion of participants designated to the smallest profile (most probable profile membership); Prob. max, maximum of the diagonal of the average latent class probabilities for most probable profile; Prob min, minimal of the diagonal of the average latent class probabilities for most probable profile; SABIC, Sample Size‐Adjusted Bayesian Information Criterion.

Latent profile solutions ranging from one to five classes were compared using multiple fit indices, as fit generally improved as more profiles were added. The four‐ and five‐profile solutions yielded marginally lower AIC, BIC, CAIC, and SABIC values than the three‐profile solution, indicating better relative model fit. Specifically, AIC decreased from 4523 (three‐profile) to 4435 (five‐profile), and BIC improved from 4646 (three‐profile) to 4624 (five‐profile). Entropy remained high across the three‐, four‐, and five‐profile solutions (0.884, 0.867, and 0.883, respectively), suggesting acceptable class separation in all three models.

Despite these improvements in relative fit, both the four‐ and five‐profile solutions yielded profiles with very small subgroup sizes (*n* < 25), raising concerns about the interpretability and stability. Moreover, visual inspection of the profile structures revealed that the additional profiles largely reflected minor variations of patterns already captured in the three‐profile solution, rather than conceptually distinct response types.

Taken together, while the four‐ and five‐profile models showed marginal statistical improvements, the three‐profile solution was selected as the most parsimonious and interpretable model with adequate class separation, conceptually meaningful patterns, and sufficient class sizes for downstream analyses.

**FIGURE 1:**
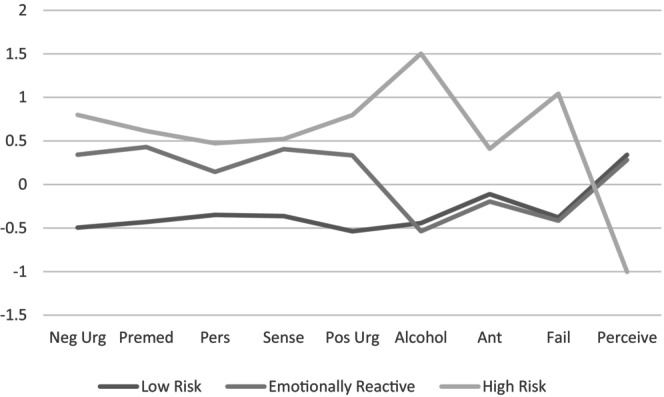
Mean comparisons of the three identified latent profiles. Neg Urg, negative urgency; Premed, lack of premediation; Pers, perseverance; Sense, sensation seeking; Pos Urg, positive urgency; Alcohol, AUDIT score; Ant, anticipated control; Fail, failed control; Perceive, perceived control.

The three profiles were identified as follows: (1) Low‐Risk group (*n* = 108; 51.9% male, 48.1% female; M_age_ = 42.24, SD = 15.14), (2) Emotionally Reactive group (*n* = 52; 41.2% male, 58.8% female; M_age_ = 32.81, SD = 9.62), and (3) High‐Risk group (*n* = 28; 57.1% male, 42.9% female; *M*_age_ = 38.54, SD = 9.57). As illustrated in Figure 1, the Low‐Risk profile exhibited low levels of both impulsivity and AUD symptoms. The Emotionally Reactive profile displayed high impulsivity but low levels of AUD symptoms, while the High‐Risk profile was characterized by high impulsivity and AUD symptoms. Table 3 presents comparisons across the three profiles in terms of AUD symptoms, anticipated control, failed control, predicted control, and UPPS‐P traits.

**TABLE 3:** Means, standard deviations, ANCOVA *F* statistic, Vovk‐Sellke maximum *p*‐ratio, and eta squared of latent profile variables.

	Mean (SD) of low risk (*n* = 113)	Mean (SD) of emotionaly reactive (*n* = 52)	Mean (SD) of high risk (*n* = 28)	Bayesian ANCOVA model	*F* statistic	Group differences	VS‐MPR	*η* ^2^	*η*_*p*_^2^
AUDIT	3.87 (3.87)	6.88 (4.70)	24.43 (6.09)	P(M|Data)_MA_ = 0.65, BF_M_ = 12.92, BF_10_ = 8.71 × 10^+49^	*F* (2, 181) = 4715.88, *p* < 0.001	1 <<< 2*** 1 <<< 3*** 2 << 3***	5.66 × 10^+49^	0.73	0.74
Anticipated control	8.20 (7.81)	8.21 (6.82)	14.11 (5.20)	P(M|Data)_M_ = 0.62, BF_M_ = 11.52, BF_10_ = 40.79	*F* (2, 181) = 7.56, *p* < 0.001	1 = 2 3 >>> 1** 3 >> 2***	72.31	0.08	0.08
Failed control	9.35 (6.15)	11.96 (6.02)	23.36 (5.93)	P(M|Data)_M_ = 0.42, BF_M_ = 5.07, BF_10_ = 5.97 × 10^+16^	*F* (2, 181) = 58.93, *p* < 0.001	2 > 1 3 >>> 1*** 3 >>> 2***	4.17 × 10^+17^	0.39	0.39
Predicted control	31.73 (9.86)	27.54 (9.36)	13.46 (6.62)	P(M|Data)_M_ = 0.71, BF_M_ = 17.41, BF_10_ = 4.29 × 10^+12^	*F* (2, 181) = 40.49, *p* < 0.001	1 = 2 1 >>> 3*** 2 >>> 3***	3.75 × 10^+12^	0.31	0.31
Negative urgency	2.38 (0.42)	3.28 (0.42)	2.98 (0.48)	P(M|Data)_MGA_ = 0.51, BF_M_ = 7.32, BF_10_ = 7.74 × 10^+18^	*F* (2, 181) = 49.37 *p* < 0.001	2 >>> 1*** 3 >>> 1*** 2 = 3	1.21 × 10^+15^	0.34	0.35
Lack of premeditation	1.84 (0.45)	2.56 (0.43)	2.17 (0.45)	P(M|Data)_M_ = 0.71, BF_M_ = 17.49, BF_10_ = 1.44 × 10^+14^	*F* (2,181) = 43.42 *p* < 0.001	2 >>> 1*** 2 >>> 3** 3 >> 1**	2.622 × 10^+13^	0.32	0.32
Lack of perseverance	2.24 (0.53)	2.71 (0.44)	2.62 (0.47)	P(M|Data)_M_ = 0.61, BF_M_ = 10.79, BF_10_ > 100	*F* (2, 181) = 16.34, *p* < 0.001	2 >>> 1*** 3 >> 1** 2 = 3	81,997	0.15	0.15
Sensation seeking	2.23 (0.61)	3.06 (0.61)	2.56 (0.57)	P(M|Data)_M_ = 0.36, BF_M_ = 3.99, BF_10_ > 100	*F* (2, 181) = 25.30, *p* < 0.001	2 >>> 1*** 2 >> 3** 3 = 1	8.05 × 10^+7^	0.21	0.22
Positive urgency	1.78 (0.56)	2.95 (0.58)	2.58 (0.77)	P(M|Data)_M_ = 0.54, BF_M_ = 8.19, BF_10_ > 100	*F* (2,181) = 57.79, *p* < 0.001	2 >>> 1*** 2 > 3 3 >>> 1***	2.12 × 10^+17^	0.38	0.39

*Note*: P(M) is 0.13 for all Bayesian ANOVAS. With respect to frequentist statistics, >Represents *p* < 0.05. >>represents *p* < 0.01. >>>represents *p* < 0.001. With respect to Bayesian statistics, ***Represents BF_10_ > 100; **Represents BF_10_ > 30; *Represents BF_10_ > 10. *η*_*p*_^2^ represents partial eta squared. ANCOVAs were performed with gender and age as covariates. Eta squared accounts for the effects of profiles without the effects of age and gender. Vovk‐Sellke Maximum *p* ‐ratio is reported using the *p*‐value; this value provides the maximal odds in favor of H₁ over H₀ equals 1/(−e *p* log(*p*)) for *p* ≤ 0.37 (Sellke et al., 2001).

No significant gender differences were observed among the profiles. However, there was strong evidence that participants in the Low‐Risk group were older than those in the Emotionally Reactive group (prior odds = 0.59; posterior odds = 187; BF_10_ = 318.3, error < 0.001). The AUDIT emerged as the most significant variable for differentiating profiles (*ηp* ^2^ = 0.74; VS‐MPR = 5.66 × 10^49^), whereas anticipated control was the least significant (*ηp* ^2^ = 0.08; VS‐MPR = 72.31). These findings suggest that AUD symptoms were the primary factor distinguishing the profiles. Additional details are provided in Table 1.

### Associations between extracted profiles and personality

With the five‐factor model of normative personality, group differences were found in neuroticism (P[M|Data]_MGA_ = 0.64, BF_M_ = 12.45, BF_10_ > 100), agreeableness (P[M|Data]_MGA_ = 0.77, BF_M_ = 23.48, BF_10_ > 100), and conscientiousness (P[M|Data]_MA_ = 0.34, BF_M_ = 3.66, BF_10_ > 100). Specifically, the Emotionally Reactive group scored higher in neuroticism (BF_10_ > 100 between group; VS‐MPR = 6.00; *ηp* ^2^ = 0.05) and lower in conscientiousness (BF_10_ > 100 between group; VS‐MPR = 36.32; *ηp* ^2^ = 0.07) than the Low‐Risk group. Both the Emotionally Reactive (BF_10_ > 30) and High‐Risk groups (BF_10_ > 10) scored lower in agreeableness than the Low‐Risk group (BF_10_ > 100 all groups; VS‐MPR = 17.09; *ηp* ^2^ = 0.06).

With pathological personality, differences were found among profiles in negative affectivity (P[M|Data]_MGA_ = 0.56, BF_M_ = 8.96, BF_10_ > 100), antagonism (P[M|Data]_MGA_ = 0.60, BF_M_ = 10.30, BF_10_ > 100), disinhibition (P[M|Data]_MA_ = 0.46, BF_M_ = 6.00, BF_10_ > 100), and psychoticism (P[M|Data]_MA_ = 0.78, BF_M_ = 24.73, BF_10_ > 100) (Table 4). Specifically, negative affectivity (VS‐MPR = 1508.74; *ηp* ^2^ = 0.11) and psychoticism were higher in the Emotionally Reactive profile than in the Low‐Risk group. Antagonism (VS‐MPR = 20,622; *ηp* ^2^ = 0.14) and disinhibition (VS‐MPR = 1.08x10^+16^; *ηp* ^2^ = 0.37) were higher in High‐Risk and Emotionally Reactive than Low Risk. With behavioral activation and inhibition, BAS Fun (VS‐MPR = 7.19; *ηp* ^2^ = 0.05) was higher in Emotionally Reactive than High‐Risk and Low Risk (P[M|Data]_M_ = 0.41, BF_M_ = 4.84, BF_10_ = 19.25).

**TABLE 4:** Means, standard deviations, ANCOVA *F* statistic, Vovk‐Sellke maximum *p*‐ratio, and eta squared of latent profiles and broad and pathological personality traits.

	Mean (SD) of low risk (*n* = 113)	Mean (SD) of emotionaly reactive (*n* = 52)	Mean (SD) of high risk (*n* = 28)	Bayesian ANCOVA model	*F* statistic	Group differences	VS‐MPR	*η*_*p*_^2^
Neuroticism	3.42 (0.71)	3.88 (0.69)	3.69 (0.66)	P(M|Data)_MGA_ = 0.64, BF_M_ = 12.45, BF_10_ > 100	*F* (2, 181) = 4.34 *p* < 0.01	2 >> 1*** 1 = 3 2 = 3	6.00	0.05
Agreeableness	4.22 (0.47)	3.89 (0.63)	3.90 (0.56)	P(M|Data)_MGA_ = 0.77, BF_M_ = 23.48, BF_10_ > 100	*F* (2, 181) = 5.73, *p* < 0.01	1 >> 2** 1 > 3* 2 = 3	17.09	0.06
Conscientiousness	3.27 (0.61)	2.86 (0.58)	2.98 (0.71)	P(M|Data)_MA_ = 0.34, BF_M_ = 3.66, BF_10_ > 100	*F* (2, 181) = 6.69, *p* < 0.01	1 >> 2*** 1 = 3 2 = 3	36.32	0.07
Extraversion	3.11 (0.84)	3.27 (0.66)	3.16 (0.56)	P(M|Data)_M_ = 0.04, BF_M_ = 0.29, BF_10_ = 0.13	*F* (2, 181) = 0.26, *p* = 0.79	1 = 2 = 3	1.00	<0.01
Openness	3.84 (0.60)	3.90 (0.57)	3.88 (0.60)	P(M|Data)_MA_ = 0.03, BF_M_ = 0.25, BF_10_ = 4.04	*F* (2, 181) = 0.13, *p* = 0.87	1 = 2 = 3	1.00	<0.01
Negative affectivity	1.45 (0.56)	2.01 (0.54)	1.74 (0.63)	P(M|Data)_MGA_ = 0.56, BF_M_ = 8.96, BF_10_ = 2.33 × 10^+7^	*F* (2,181) = 11.34, *p* < 0.001	2 >>> 1*** 3 > 1 2 = 3	1508.74	0.11
Detachment	1.29 (0.63)	1.39 (0.60)	1.46 (0.49)	P(M|Data)_M_ = 0.09, BF_M_ = 0.69, BF_10_ = 0.15	*F* (2, 181) = 1.30, *p* = 0.28	1 = 2 = 3	1.04	0.01
Antagonism	0.54 (0.41)	1.07 (0.60)	0.89 (0.46)	P(M|Data)_MGA_ = 0.60, BF_M_ = 10.30, BF_10_ = 7.66 × 10^+7^	*F* (2,181) = 14.61, *p* < 0.001	2 >>> 1*** 3 >> 1** 2 = 3	20,622	0.14
Disinhibition	0.94 (0.41)	1.72 (0.39)	1.50 (0.55)	P(M|Data)_MA_ = 0.46, BF_M_ = 6.00, BF_10_ = 4.61 × 10^+19^	*F* (2,181) = 52.89, *p* < 0.001	2 >>> 1*** 3 >>> 1*** 2 = 3	1.08x10^+16^	0.37
Psychoticism	0.75 (0.50)	1.31 (0.64)	0.98 (0.52)	P(M|Data)_MA_ = 0.78, BF_M_ = 24.73, BF_10_ = 2.14 × 10^+7^	*F* (2, 181) = 12.38, *p* < 0.001	2 >>> 1*** 1 = 3 2 = 3	3464.20	0.12
BIS	22.32 (4.24)	22.48 (3.68)	20.96 (4.15)	P(M|Data)_MA_ = 0.35, BF_M_ = 3.75, BF_10_ = 1.19	*F* (2, 181) = 1.73, *p* = 0.18	1 = 2 = 3	1.19	0.02
BAS reward	16.42 (2.35)	16.67 (2.04)	15.50 (2.77)	P(M|Data)_MGA_ = 0.14, BF_M_ = 1.18, BF_10_ = 4.89	*F* (2, 181) = 2.14, *p* = 0.12	1 = 2 = 3	1.44	0.02
BAS drive	9.83 (3.04)	10.46 (2.12)	9.75 (3.24)	P(M|Data)_M_ = 0.05, BF_M_ = 0.33, BF_10_ = 0.17	*F* (2, 181) = 0.39, *p* = 0.68	1 = 2 = 3	1.00	<0.01
BAS fun	10.50 (2.72)	12.02 (2.07)	10.46 (2.32)	P(M|Data)_M_ = 0.41, BF_M_ = 4.84, BF_10_ = 19.25	*F* (2,181) = 4.58, *p* < 0.05	2 > 1** 2 > 3* 1 = 3	7.19	0.05

*Note*: P(M) is 0.13 for all Bayesian ANOVAS. With respect to frequentist statistics, >Represents *p* < 0.05; >>Represents *p* < 0.01; >>>Represents *p* < 0.001. With respect to Bayesian statistics, ***Represents BF_10_ > 100; **Represents BF_10_ > 30; *Represents BF_10_ > 10. *η*_*p*_^2^ represents partial eta squared. ANCOVAs were performed with gender and age as covariates. Eta squared accounts for the effects of profiles without the effects of age and gender. Vovk‐Sellke Maximum *p* ‐ratio is reported using the *p*‐value; this value provides the maximal odds in favor of H₁ over H₀ equals 1/(−e *p* log(*p*)) for *p* ≤ 0.37 (Sellke et al., 2001).

### Associations between extracted profiles and behavioral measures

Group differences were evident in PRT Discriminability (P[M|Data]_M_ = 0.57, BF_M_ = 9.29, BF_10_ > 100) and PRT accuracy (P[M|Data]_MA_ = 0.43, BF_M_ = 5.28, BF_10_ > 100). Specifically, the Low‐Risk profile outperformed the High‐Risk group in PRT accuracy (VS‐MPR = 3.37; *ηp* ^2^ = 0.04). Moreover, PRT discriminability scores (VS‐MPR = 6.08; *ηp* ^2^ = 0.05) revealed both Low‐Risk and Emotionally Reactive groups outperformed the High‐Risk group. Otherwise, PRT reaction time and response bias are equivalent across profiles. Profile membership did not predict BART, GNG, or SSRT scores (Table 5).

**TABLE 5:** Means, standard deviations, ANCOVA *F* statistic, Vovk‐Sellke maximum *p*‐ratio, and eta squared of latent profiles.

	Mean (SD) of low risk (*n* = 113)	Mean (SD) of emotionaly reactive (*n* = 52)	Mean (SD) of high risk (*n* = 28)	Bayesian ANCOVA model	*F* statistic	Group differences	VS‐MPR	*η*_*p*_^2^
BART adjusted average pump	28.43 (15.57)	27.90 (14.53)	29.12 (14.33)	P(M|Data)_M_ = 0.03, BF_M_ = 0.25, BF_10_ = 0.07	*F* (2, 181) = 0.17, *p* = 0.84	1 = 2 = 3	1.00	<0.01
BART total explosions	7.21 (4.49)	6.88 (3.97)	7.18 (4.70)	P(M|Data)_M_ = 0.04, BF_M_ = 0.26, BF_10_ = 0.07	*F* (2, 181) = 0.33, *p* = 0.72	1 = 2 = 3	1.00	<0.01
GNG RT	402.98 (100.17)	362.13 (52.42)	369.69 (52.86)	P(M|Data)_MA_ = 0.15, BF_M_ = 1.22, BF_10_ = 4117	*F* (2,181) = 1.47, *p* = 0.23	1 = 2 = 3	1.08	0.02
SST SSRT	217.61 (56.60)	222.52 (56.38)	216.44 (62.68)	P(M|Data)_MA_ = 0.09, BF_M_ = 0.66, BF_10_ = 0.77	*F* (2, 181) = 0.90, *p* = 0.41	1 = 2 = 3	1.00	<0.01
WAIS MR scaled	10.92 (3.32)	11.08 (3.02)	10.11 (3.49)	P(M|Data)_M_ = 0.14, BF_M_ = 0.33, BF_10_ = 0.13	*F* (2, 182) = 0.98, *p* = 0.38	1 = 2 = 3	1.00	0.01
WAIS VC scaled	12.03 (3.26)	12.27 (5.60)	11.71 (2.75)	P(M|Data)_M_ = 0.06, BF_M_ = 0.20, BF_10_ = 0.08	*F* (2, 183) = 0.18, *p* = 0.84	1 = 2 = 3	1.00	<0.01
PRT RT	674.74 (238.48)	593.55 (174.11)	665.63 (258.30)	P(M|Data)_MGA_ = 0.04, BF_M_ = 0.30, BF_10_ = 6.89 × 10^+7^	*F* (2, 169) = 0.13, *p* = 0.88	1 = 2 = 3	1.00	<0.01
PRT accuracy	0.80 (0.10)	0.83 (0.11)	0.75 (0.11)	P(M|Data)_M_ = 0.43, BF_M_ = 5.28, BF_10_ = 347.54	*F* (2,169) = 3.53, *p* < 0.05	1 = 2 1 > 3 2 = 3	3.37	0.04
PRT response bias	0.12 (0.33)	0.10 (0.17)	0.20 (0.41)	P(M|Data)_M_ = 0.08, BF_M_ = 0.64, BF_10_ = 0.15	*F* (2, 169) = 1.06, *p* = 0.35	1 = 2 = 3	1.00	0.01
PRT discriminability	0.69 (0.30)	0.78 (0.35)	0.53 (0.27)	P(M|Data)_M_ = 0.57, BF_M_ = 9.29, BF_10_ = 355.41	*F* (2, 169) = 4.36, *p* < 0.05	1 = 2 1 > 3 2 > 3*	6.08	0.05

*Note*: P(M) is 0.13 for all Bayesian ANOVAS. With respect to frequentist statistics, >Represents *p* < 0.05; >>Represents *p* < 0.01; >>>Represents *p* < 0.001. With respect to Bayesian statistics, Represents BF_10_ > 100; Represents BF_10_ > 30; *Represents BF_10_ > 10. *η*_*p*_^2^ represents partial eta squared. ANCOVAs were performed with gender and age as covariates. Eta squared accounts for the effects of profiles without the effects of age and gender. Vovk‐Sellke Maximum *p* ‐ratio is reported using the *p*‐value; this value provides the maximal odds in favor of H₁ over H₀ equals 1/(−e *p* log(*p*)) for *p* ≤ 0.37 (Sellke et al., 2001).

### Associations between extracted profiles and DSM diagnoses

Table 6 displays the number of cases and standardized residuals of profile membership and diagnosis. Profile membership was significantly associated with an anxiety disorder diagnosis (χ^2^[2, *N* = 201] = 6.93, *p* < 0.05). Specifically, the High‐Risk profile was more likely to meet criteria for a lifetime anxiety disorder (*z* = 2.51). Significant relationships were found between profile membership and lifetime substance use disorder (χ^2^[2, *N* = 201] = 35.50, *p* < 0.001) with the Low‐Risk group (*z* = −5.54) less likely and the Emotionally Reactive (*z* = 2.30) and High‐Risk (*z* = 4.83) groups more likely to meet criteria. Significant associations were found between profile membership and mood disorder (χ^2^[2, *N* = 200] = 11.68, *p* < 0.01) as the Low‐Risk profile was more likely to have met criteria for a lifetime mood disorder while the Emotionally Reactive group was less likely to have met criteria.

**TABLE 6:** Chi‐square statistics for DSM diagnoses across profiles.

	Mean (SD) of low risk (*n* = 113)	Mean (SD) of emotionally reactive (*n* = 52)	Mean (SD) of high risk (*n* = 28)
Anxiety disorder (Lifetime)^a^			
Absent	59 (0.56)	30 (1.38)	8 (−2.51)
Present	54 (−0.56)	21 (−1.38)	20 (2.51)*
Anxiety disorder (Current)			
Absent	82 (1.90)	33 (−0.44)	14 (−2.10)
Present	31 (−1.90)	18 (0.44)	14 (2.10)*
Substance use disorder (Lifetime)^a^			
Absent	69 (5.48)	16 (−2.34)	1 (−4.72)
Present	44 (−5.48)	36 (2.34)*	27 (4.72)*
Substance use disorder (Current)^a^			
Absent	104 (5.54)	36 (−2.30)	13 (−4.83)
Present	7 (−5.54)	16 (2.30)*	14 (4.83)*
Mood disorder (Lifetime)^a^			
Absent	21 (−3.34)	22 (2.90)*	10 (1.04)
Present	92 (3.34)*	29 (−2.90)	18 (−1.04)
Mood disorder (Current)			
Absent	73 (−0.91)	37 (0.95)	19 (0.08)
Present	40 (0.91)	14 (−0.95)	9 (−0.08)

*Note*: Number represents number of participants meeting or not meeting criteria for DSM diagnoses. Number in brackets represents the standardized residual. The standardized residuals are computed by (observed ‐ expected) / sqrt(expected × [1 ‐ row marginal proportion] × [1 ‐ column marginal proportion]).

^a^ Represents *p* < 0.05 in chi‐square tests.

* Represents > 1.96 in standard residuals.

## DISCUSSION

This study examined latent profiles derived from measures of impulsivity and alcohol use disorder symptoms, and explored their associations with performance‐based impulsivity tasks and self‐reported personality traits. In addressing the first research question, three distinct profiles were identified: Low Risk, Emotionally Reactive, and High Risk, representing groups with varying levels of alcohol consumption causing distress and impairment along with impulsivity. Consistent with previous research, impulsivity was more prevalent among younger individuals, although no significant gender differences were observed, despite males typically reporting higher impulsivity (Chamorro et al., 2012). While the analysis identified an impulsive profile with lower AUDIT scores, no profile was found to exhibit high AUDIT scores with low impulsivity scores. These findings align with prior research emphasizing the central role of impulsivity in self‐regulatory and motivational processes underlying substance use disorders (Lee et al., 2019; Stamates et al., 2023).

In relation to the second research question, distinct associations were observed between profile membership and personality traits. While no significant differences were noted in extraversion or openness to experience, the Emotionally Reactive group exhibited higher levels of neuroticism and lower levels of conscientiousness compared to the Low‐Risk group. This aligns with findings by Mao et al. (2018), which suggest that neuroticism and conscientiousness share substantial conceptual overlap with self‐control, potentially mediating the relationship between impulsivity and broad personality traits. The Emotionally Reactive group may also reflect individuals prone to error‐related negativity, a neurobehavioral trait characterized by negative affect, impulsivity, and conscientiousness, which is predictive of abnormal error processing (Hill et al., 2016).

Both the Emotionally Reactive and High‐Risk groups showed lower levels of agreeableness and higher levels of antagonism and disinhibition compared to the Low‐Risk group. These findings are consistent with prior research indicating that impulsive individuals are at greater risk for externalizing psychopathologies beyond substance use behaviors (Carver & Johnson, 2018). Additionally, the Emotionally Reactive group scored higher in fun seeking compared to both High‐Risk and Low‐Risk profiles. Fun seeking may reflect a tendency toward pleasure, disinhibition, and novelty‐seeking behaviors not fully captured by the BAS subscales alone (Carver & White, 1994; Cloninger, 1987; Watson & Clark, 1993). This is consistent with evidence linking fun seeking to noncomorbid alcohol use disorders, particularly among individuals without cooccurring anxiety disorders (Conrod et al., 2000; Johnson et al., 2003).

The tension‐reduction hypothesis suggests that individuals may use substances to alleviate anxiety, potentially leading to comorbid substance use and anxiety disorders (Young et al., 1990). Since the High‐Risk group demonstrated a greater likelihood of meeting criteria for an anxiety disorder, it may represent individuals particularly vulnerable to the cooccurrence of anxiety and substance use disorders.

Regarding the third research question, no significant differences were observed among the profiles on performance‐based impulsivity tasks, except for measures of PRT accuracy and discriminability. Specifically, both the Low‐Risk and Emotionally Reactive groups outperformed the High‐Risk group in discriminability, which serves as an indicator of task difficulty and participant performance in distinguishing between two stimuli (Pizzagalli et al., 2008). Additionally, the Low‐Risk group demonstrated higher accuracy than the High‐Risk group. Accuracy, a secondary variable, reflects the participant's hit rate and is associated with abnormal reward responsiveness (Pizzagalli et al., 2008). These findings suggest that individuals with AUD symptoms may be particularly susceptible to reward‐based impulsivity, potentially increasing their risk for hazardous drinking behaviors (Stamates & Lau‐Barraco, 2017). Alternatively, habitual heavy alcohol consumption may impair reward processing and other cognitive functions, contributing to the observed performance deficits. To better understand these associations, future research should consider employing longitudinal designs to explore the causal relationships between alcohol use, impulsivity, and cognitive performance.

### Clinical implications

The identification of distinct latent profiles, Low Risk, Emotionally Reactive, and High Risk, underscores the heterogeneity in impulsivity and alcohol use patterns. This differentiation highlights the need for early intervention strategies tailored to specific risk profiles. For example, individuals who exhibit high impulsivity but low alcohol consumption may benefit from preventive interventions that focus on enhancing self‐regulation and coping skills, reducing the likelihood of developing hazardous drinking behaviors. Early interventions such as Screening, Brief Intervention, and Referral to Treatment (SBIRT) have demonstrated efficacy in reducing substance use and related harms when implemented in primary care settings (Babor et al., 2007).

Furthermore, the Emotionally Reactive group's elevated neuroticism and reduced conscientiousness suggest a predisposition to emotion‐driven impulsivity, which has been associated with an increased risk of substance use (Whiteside & Lynam, 2001). The present findings show that behavioral activation in fun seeking was higher in Emotionally Reactive than High Risk and Low Risk. Tailored interventions targeting emotion regulation difficulties, such as cognitive‐behavioral therapies, could help mitigate this risk. In contrast, the High‐Risk group's lower discriminability and accuracy on performance‐based tasks point to potential deficits in reward processing, consistent with research showing that impaired decision making and reward sensitivity are linked to substance use disorders (Bechara, 2005). Interventions that address these cognitive deficits, including cognitive remediation therapies, may improve treatment outcomes for this subgroup.

Importantly, incorporating personality assessments into treatment planning can guide the selection of therapeutic approaches that align with an individual's unique risk profile. For example, individuals with high sensation‐seeking traits may respond more favorably to interventions that incorporate novelty and stimulation, consistent with their personality style (Conrod et al., 2000). This person‐centered approach to treatment planning, which accounts for individual differences in personality and cognitive functioning, is in line with the broader movement toward personalized medicine in mental health care (Insel, 2009).

### Limitations and summary

This study has some limitations. First, it is important to separate impulsivity traits as risk factors as opposed to consequences for alcohol use (Herman & Duka, 2019). The cross‐sectional design and small sample size limit the ability to infer causal relationships between alcohol use, impulsivity, and cognitive performance. A longitudinal design was not feasible due to resource and time constraints, but future research using this approach would offer valuable insights into the directionality and temporal dynamics of these associations. Future studies with larger samples will be important for determining whether more nuanced class structures are replicable and substantively meaningful. Second, impulsivity may be conceptualized based on self‐report and impulsivity‐related constructs studied in the laboratory (i.e., inattention, inhibition, impulsive decision making, and shifting; Stevens et al., 2018). Future studies would benefit from including all these aspects of impulsivity in the LPA to determine whether it may distinguish additional profiles related to risk for problematic alcohol use.

In addition, a key limitation is that psychiatric comorbidities were not included as profile indicators during the latent profile analysis. While these diagnoses were considered in later profile comparisons, their exclusion from the initial model may have limited the identification of clinically distinct subgroups. Given that psychiatric conditions can influence both impulsivity traits and alcohol‐related outcomes, their presence may confound profile structure and interpretation. Future research should consider integrating diagnostic variables into the profile derivation process or using stratified approaches to better account for heterogeneity due to comorbidity. Lastly, impulsivity may manifest differently across situations. For instance, agreeableness is associated with interpersonal impulsivity, whereas conscientiousness is linked with schoolwork impulsivity (Tsukayama et al., 2013). Hence, future studies may investigate whether situational‐dispositional representations of personality traits may provide greater insight. Despite these limitations, the current investigation provided insight into differential groups of individuals at risk of AUD symptoms and impulsivity. Future studies may employ these findings to determine whether these profiles may be predictive of treatment outcomes and relapse.

In summary, this study offers novel insights into the heterogeneity of impulsivity and alcohol use by identifying distinct latent profiles within a clinically enriched population. Through the integration of self‐report, clinician‐administered, and performance‐based data, the analysis revealed meaningful differences in personality traits, cognitive performance, and psychiatric diagnoses across subgroups. The identification of an emotionally reactive but non‐drinking group challenges the assumption that impulsivity uniformly leads to alcohol‐related harm, highlighting the need for tailored interventions based on specific risk profiles. These findings underscore the value of person‐centered, transdiagnostic approaches in understanding impulsivity‐related psychopathology and lay the groundwork for future longitudinal and clinical research to enhance the precision of prevention and treatment strategies for individuals at risk of substance use disorders.

## CONFLICT OF INTEREST STATEMENT

The authors declared no potential conflicts of interest with respect to the research, authorship, and/or publication of this article.

## ETHICS STATEMENT

The study was approved by the CAMH Research Ethics Board. All procedures performed in studies involving human participants were in accordance with the ethical standards of Canadian National and International Guidelines as well as provincial legislation and hospital policies and with the 1964 Helsinki Declaration and its later amendments or comparable ethical standards.

## INFORMED CONSENT

This study was conducted according to the American Psychological Association (APA) and National Association of Psychology ethical standards for the treatment of human subjects. Identifying information was separated from the dataset upon analysis. Participants were informed that their participation was voluntary, that they could leave the study at any time, and that their data would be treated anonymously. Oral and written informed consent was obtained and documented by a research team member.

## Data Availability

The data presented in this study are available on request from the last author, Dr. Lena C. Quilty. The data are not publicly available.
